# A survivor with unexplained chest scars

**DOI:** 10.1186/s12245-024-00618-0

**Published:** 2024-03-28

**Authors:** Viviane Donner, Mathieu Affaticati, Elodie Izydorczyk, Sara Cereghetti

**Affiliations:** 1 Division of Intensive Care, Department of Anaesthesiology, Clinical Pharmacology, Intensive Care and Emergency Medicine, Geneva University Hospital, Rue Gabrielle-Perret-Gentil 4, Geneva, CH 1211 Switzerland; 2 Division of Emergency Medicine, Department of Anaesthesiology, Clinical Pharmacology, Intensive Care and Emergency Medicine, Geneva University Hospital, Rue Gabrielle-Perret-Gentil 4, Geneva, CH 1211 Switzerland

**Keywords:** Cardiopulmonary resuscitation, Chest compression device, PICS (Post Intensive Care Syndrome), Keloid scar

## Abstract

This case illustrates chest scars after piston-based chest compression device resuscitation and raises the awareness of the potential benefits of following up survivors of critical illness.

## Case presentation

**Fig. 1:**
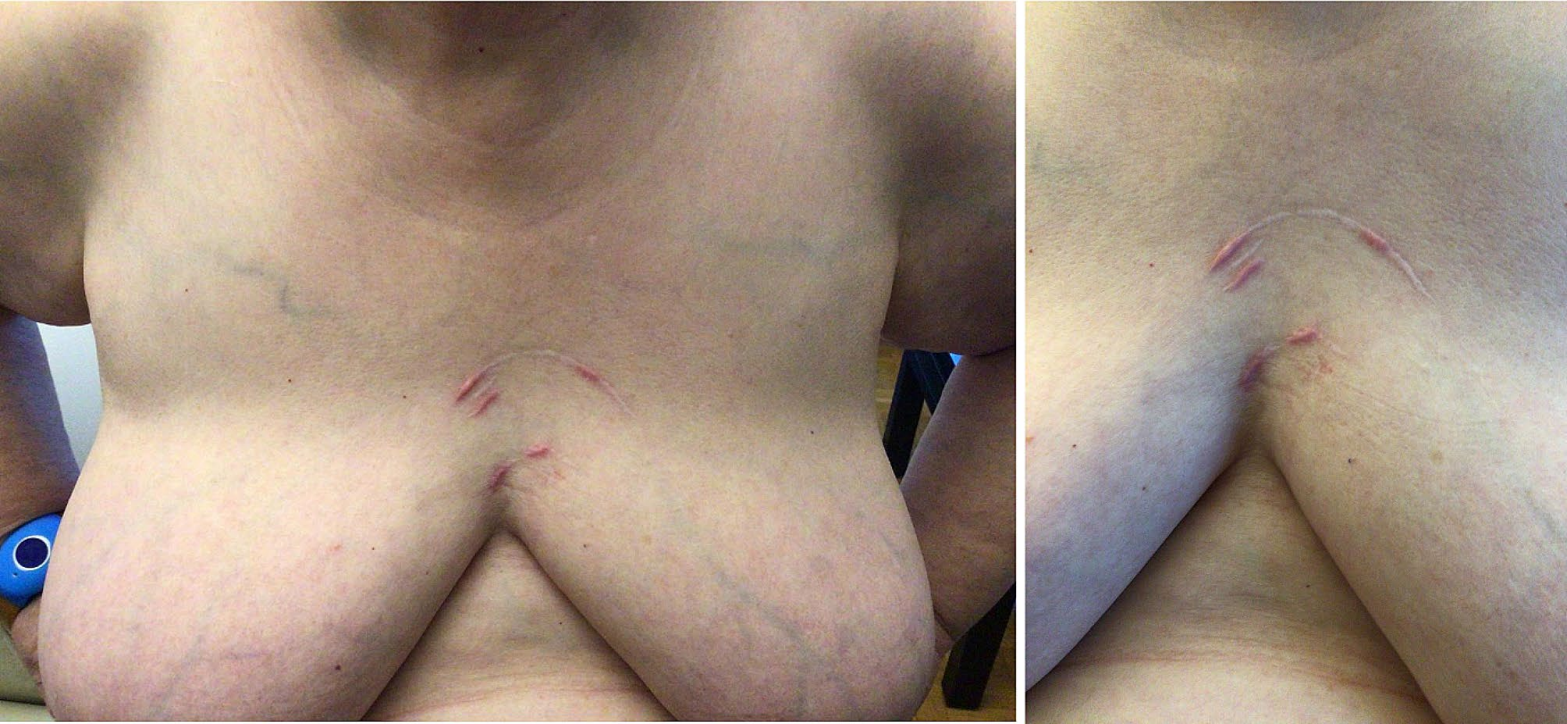
Anterior chest scars, located over the mid sternum

A 68-year-old woman enquires about the origin of painless circular scars over her anterior chest (Fig. 1) at the post ICU consultation. Seven months earlier, she was admitted into the Emergency Department for refractory cardiac arrest caused by a pulmonary embolism. She received intravenous thrombolysis, extracorporeal cardiopulmonary resuscitation (ECPR) and a total of 42 min of CPR.

## Diagnosis

*Keloid scar following CPR with a piston-based device and post-intensive care syndrome (PICS)*.

Upon examination, the keloid scars exhibit the size and circular pattern typical of the suction cup of a piston-based chest compression device. A review of the medical chart confirms that a LUCAS®3 was used in the ED to provide CPR. As previously reported in one post-mortem study, piston-based chest compression devices are associated with a higher rate of anterior chest lesions, such as hematomas and skin abrasions, compared to manual compression [1]. When using these devices, skin protection is however not recommended to avoid losing the suction effect and ensuring optimal performance.

The patient exhibits a cosmetic sequela as one element of a post intensive care syndrome (PICS). PICS encompasses new or worsening long-lasting physical, cognitive and mental health issues resulting from critical illness and adversely affecting quality of life in survivors [2]. Post-ICU follow-up programs serve to identify and address these issues.

Local steroids and laser therapy are cosmetic options to treat keloid scars. Surgical excision is usually avoided due to a high risk of recurrence. In this case, the patient was relieved to understand the origin of the scars and declined dermatological referral.

## Data Availability

Not applicable.
